# Supplementary figures and images for: Reward expectation yields distinct effects on sensory processing and decision making in the human brain
Source: PLoS Biol. 2025 Jul 7;23(7):e3003234. doi: 10.1371/journal.pbio.3003234 (PMC12251098; doi:10.1371/journal.pbio.3003234)

**A**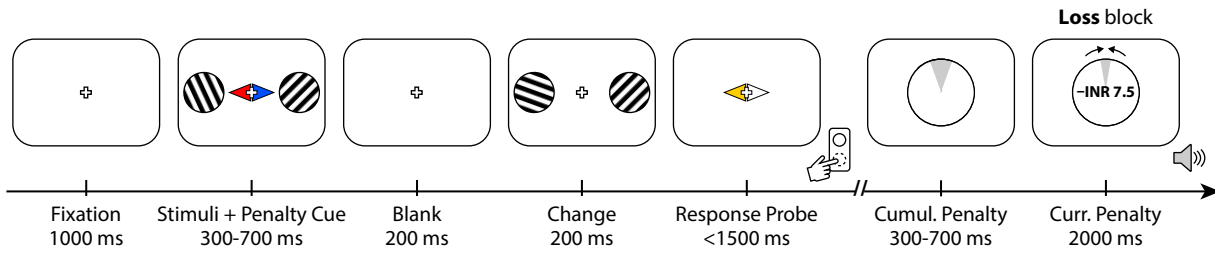**B**

Space-specific penalty expectation

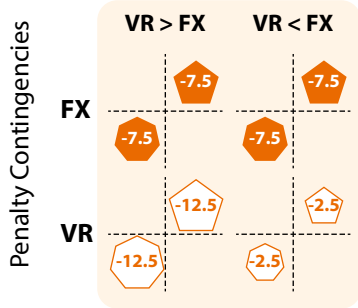**G**

Choice-specific penalty expectation

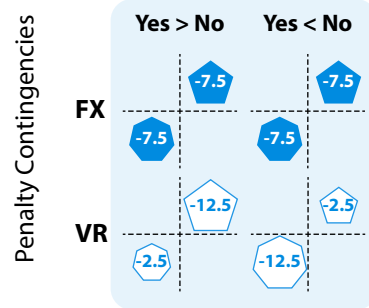**C**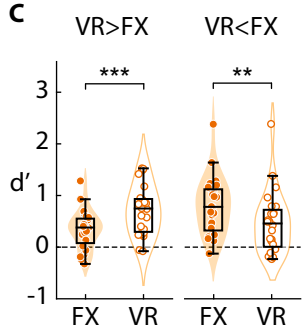**D**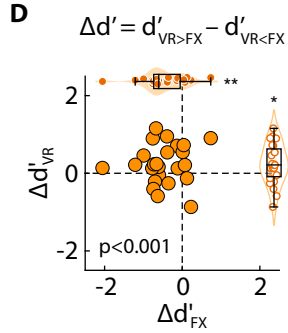**H**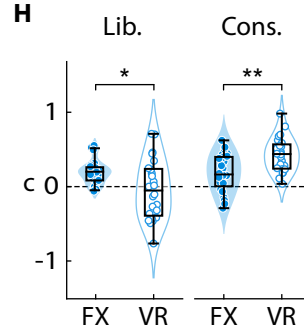**I**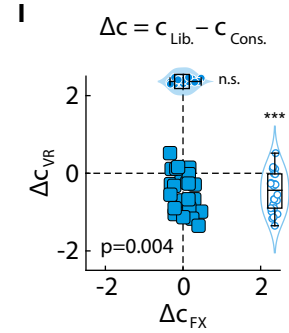**E**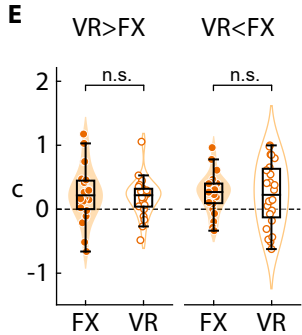**F**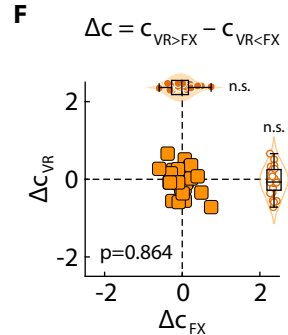**J**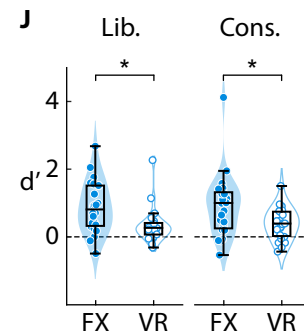**K**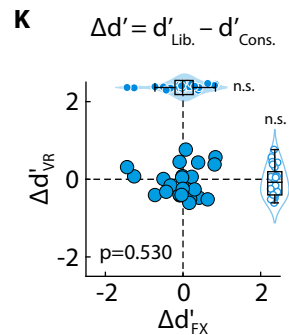

Supplement: S1 Fig — A. Same as in Fig 1A (main text), but showing the task schematic of a “loss” block trials. In these blocks participants received a penalty for incorrect responses, but no reward for correct responses. Other conventions are the same as in main Fig 1A. B. Same as in Fig 1D (main text) but showing penalty contingencies for the space-specific penalty expectation session (“loss” blocks only). Row and column conventions are the same as in Fig 1D. Numbers within polygons indicate the penalty (in INR, deducted from a baseline remuneration) for the respective, incorrect response type (FA and M). Blanks represent no reward for the correct response types (H and CR). Other conventions are the same as in main Fig 1D. (C–F). Same as in Fig 2A–2D (main text), but showing sensitivity (d′), criteria (c) and their modulations for loss block trials in the space-specific penalty expectation sessions. Other conventions are the same as in Fig 2A–2D, respectively. G. Same as in Fig 1G (main text) but showing penalty contingencies for the choice-specific penalty expectation session (“loss” blocks only). Row and column conventions are the same as in Fig 1G. Other conventions are the same as in panel B and main Fig 1G. (H–K). Same as in Fig 2E–2H (main text), but showing sensitivity (d′), criterion (c) and their modulations for loss block trials in the choice-specific penalty expectation sessions. Other conventions are the same as in Fig 2E–2H, respectively. Data are available at https://doi.org/10.6084/m9.figshare.25966015 [34]. (PDF) [file pbio.3003234.s001.pdf]

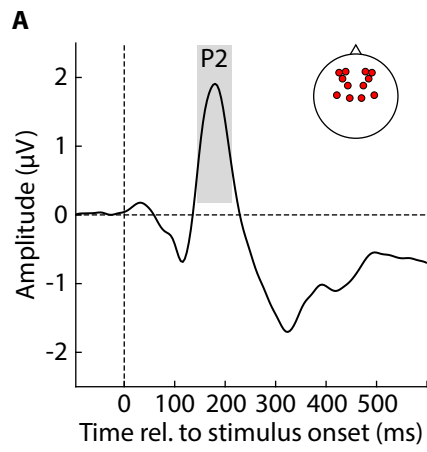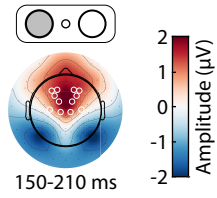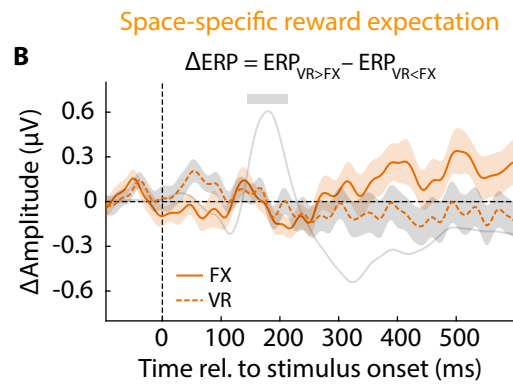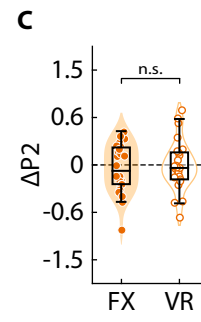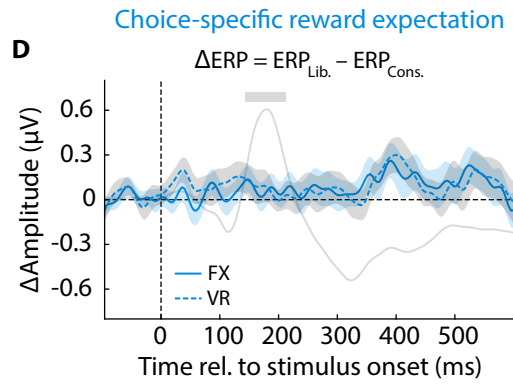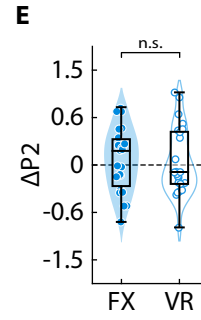

Supplement: S2 Fig — A. Top: Same as in Fig 3A (main text) but for ERP waveform measured from frontocentral electrodes (see inset) in the space-specific reward expectation session (data averaged across all n = 24 participants). Shaded regions: time epochs used to quantify anterior P2 event-related potential (grey shading). Below: Frontocentral electrodes (white circles) shown on a scalp map from which the ERP was estimated. (B–C). Same as in Fig 3B and 3C (main text), but for the contralateral P2 component measured during the space-specific reward expectation session. Other conventions are the same as in main Fig 3B and 3C. (D–E). Same as in Fig 3D and 3E (main text), but for the contralateral P2 component measured during the choice-specific reward expectation session. Other conventions are the same as in main Fig 3D–3E. Data are available at https://doi.org/10.6084/m9.figshare.25966015 [34]. (PDF) [file pbio.3003234.s002.pdf]
