# Supplementary material for: Reward expectation yields distinct effects on sensory processing and decision making in the human brain
Source: PLoS Biol. 2025 Jul 7;23(7):e3003234. doi: 10.1371/journal.pbio.3003234 (PMC12251098; doi:10.1371/journal.pbio.3003234)
Supplement: S3 Fig — A. Same as in Fig 4A (main text) but showing the reward-induced modulation of contralateral alpha power before stimulus onset (vertical dashed line). Other conventions are the same as in Fig 4A. (B–C). Same as in Fig 4B and 4C (main text) but showing pre-stimulus Δα distribution over electrodes, and their mean values in the posterior electrodes (see SI Methods), in the space-specific reward expectation session. Other conventions are the same as in Fig 4B and 4C. D. Same as in panel A, but for the choice-specific reward expectation session. Other conventions are the same as in panel A and main Fig 4A. (E–F). Same as in panels B–C, but for the choice-specific reward expectation sessions. Other conventions are the same as in panels B–C and main Fig 4E and 4F. G. Same as in Fig 4A (main text) but showing the modulation of alpha power contralateral to the VR side between high-bias and low-bias choices in the choice-specific reward expectation session. x-axis: time relative to change onset. y-axis: frequency (5–15 Hz). Other conventions are the same as in main Fig 4A. Data are available at https://doi.org/10.6084/m9.figshare.25966015 [34]. (PDF) [file pbio.3003234.s003.pdf]

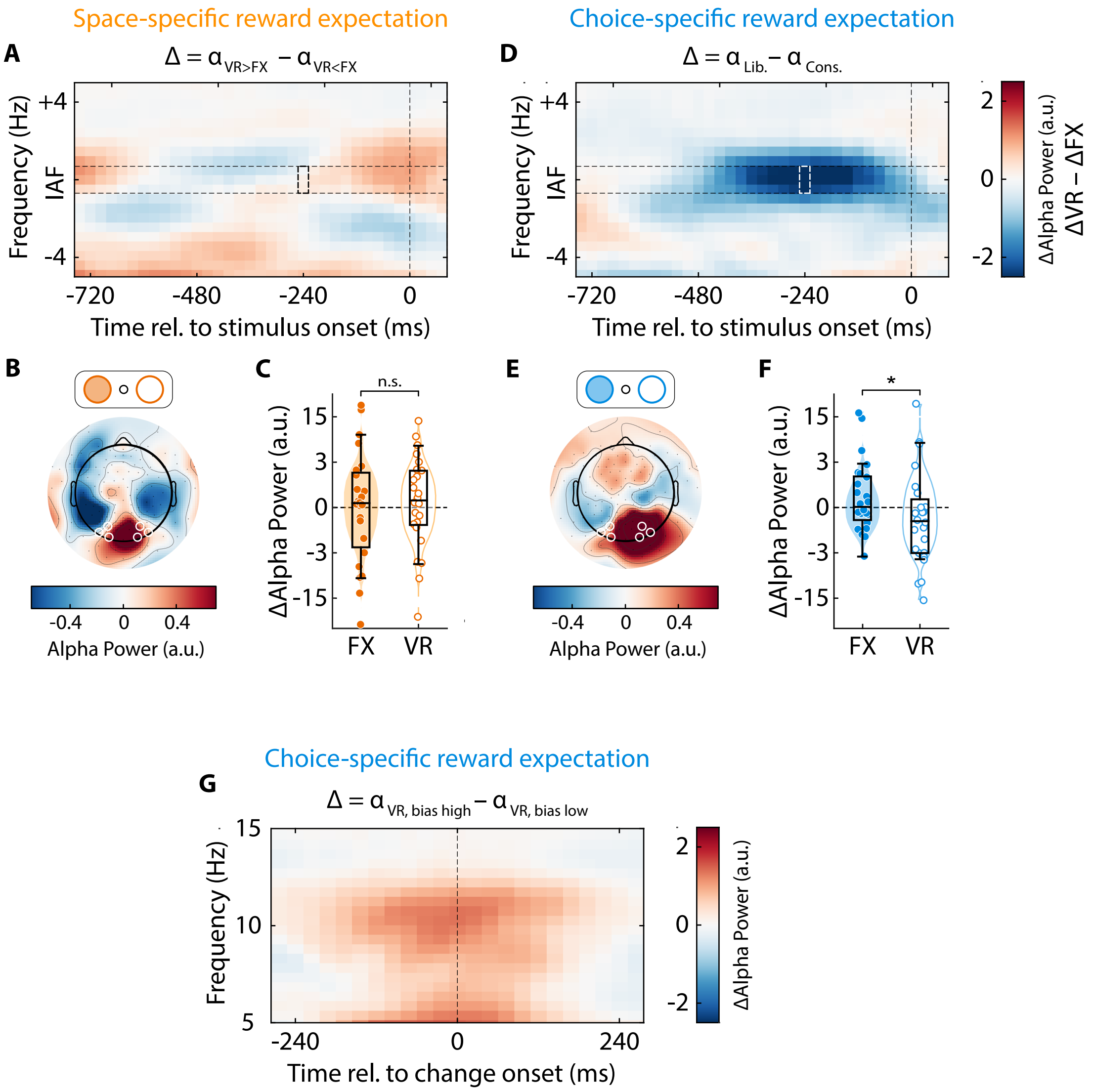

Space-specific reward expectation
Choice-specific reward expectation
A
Δ = α VR>FX − α VR<FX
Frequency (Hz)
IAF
+4
-4
-720
-480
-240
0
Time rel. to stimulus onset (ms)
D
Δ = α Lib. − α Cons.
2
0
-2
ΔAlpha Power (a.u.)
ΔVR − ΔFX
B
-0.4
0
0.4
Alpha Power (a.u.)
C
n.s.
ΔAlpha Power (a.u.)
15
3
0
-3
-15
FX
VR
E
F
*
G
Choice-specific reward expectation
Δ = α VR, bias high − α VR, bias low
Frequency (Hz)
15
10
5
-240
0
240
Time rel. to change onset (ms)
