# Supplementary material for: Reward expectation yields distinct effects on sensory processing and decision making in the human brain
Source: PLoS Biol. 2025 Jul 7;23(7):e3003234. doi: 10.1371/journal.pbio.3003234 (PMC12251098; doi:10.1371/journal.pbio.3003234)
Supplement: S1 Table — A. Reward-induced modulation of psychophysical parameters (Δd′, Δc) on each side (FX, column 3, and VR, column 4) separately in the gain (reward) and loss (penalty) blocks, as well as with the data pooled across both block types (respective, individual rows), of the space-specific reward or penalty expectation sessions. Column 5: p-values for a two-tailed significance test (signed rank). Column 6: two-sided bayes factor. B. Same as in A but for the choice-specific reward or penalty expectation sessions. (PDF) [file pbio.3003234.s004.pdf]

**A.** Space-specific reward/penalty expectation session					
		**FX**	**VR**	**p-value**	**BF_{10}**
**Δd'**	**Gain**	-0.60±0.12	0.43±0.09	< 0.001	$>10^3$
	**Loss**	-0.47±0.11	0.24±0.10	< 0.001	$>10^2$
	**Pooled**	-0.51±0.08	0.34±0.07	< 0.001	$>10^4$
**Δc**	**Gain**	0.01±0.09	0.10±0.08	0.511	0.24
	**Loss**	-0.01±0.06	-0.05±0.07	0.864	0.22
	**Pooled**	0.01±0.07	0.02±0.06	0.607	0.22

**B.** Choice-specific reward/penalty expectation session					
		**FX**	**VR**	**p-value**	**BF_{10}**
**Δd'**	**Gain**	0.05±0.09	0.15±0.10	0.391	0.29
	**Loss**	-0.04±0.11	-0.06±0.08	0.530	0.22
	**Pooled**	0.00±0.07	0.04±0.06	0.587	0.23
**Δc**	**Gain**	-0.07±0.05	-0.46±0.08	0.001	$>10^2$
	**Loss**	0.01±0.05	-0.47±0.10	0.004	25.69
	**Pooled**	-0.03±0.03	-0.46±0.08	< 0.001	$>10^2$
