# Supplementary material for: Reward expectation yields distinct effects on sensory processing and decision making in the human brain
Source: PLoS Biol. 2025 Jul 7;23(7):e3003234. doi: 10.1371/journal.pbio.3003234 (PMC12251098; doi:10.1371/journal.pbio.3003234)
Supplement: S2 Table — A. Same as in Table 1A but showing F-statistics and p-values for an ANOVA on the amplitude of P2 potentials (row 3) – in the space-specific reward expectation session. B. Same as in A but for the choice-specific reward expectation session (row 4). (PDF) [file pbio.3003234.s005.pdf]

	Side		Rew. Contingency		Side x Rew. Contingency	
	**$F_{1,23}$**	**p**	**$F_{1,23}$**	**p**	**$F_{1,23}$**	**p**
**A.** Space-specific rew. exp.	0.23	0.633	0.26	0.617	0.62	0.440
**B.** Choice-specific rew. exp.	0.97	0.335	0.00	0.953	0.04	0.835
